# Focus on Key Issues in Immune Thrombotic Thrombocytopenic Purpura: Italian Experience of Six Centers

**DOI:** 10.3390/jcm10235702

**Published:** 2021-12-04

**Authors:** Giovanni Tiscia, Maria Teresa Sartori, Gaetano Giuffrida, Angelo Ostuni, Nicola Cascavilla, Daniela Nicolosi, Cosima Battista, Teresa Maria Santeramo, Lorella Melillo, Giulio Giordano, Filomena Cappucci, Lucia Fischetti, Elena Chinni, Giuseppe Tarantini, Anna Cerbo, Antonella Bertomoro, Fabrizio Fabris, Elvira Grandone

**Affiliations:** 1 Thrombosis and Hemostasis Unit, Fondazione IRCCS Casa Sollievo della Sofferenza, 71013 San Giovanni Rotondo, Italy; g.tiscia@operapadrepio.it (G.T.); f.cappucci@operapapdrepio.it (F.C.); l.fischetti@operapadrepio.it (L.F.); e.chinni@operapadrepio.it (E.C.); 2 Department of Internal Medicine, University of Padua, 35100 Padua, Italy; mtsart@unipd.it (M.T.S.); emostasi1@operapadrepio.it (A.C.); antonella.bertomoro@aopd.veneto.it (A.B.); fabrizio.fabris@unipd.it (F.F.); 3 Hematology Division, Department of Clinical and Molecular Biomedicine, University of Catania, 95123 Catania, Italy; gaegiuffrida@gmail.com (G.G.); danielanicolosi03@gmail.com (D.N.); 4 Transfusion Medicine & Blood Bank, University-Hospital of Bari, 70124 Bari, Italy; angelo.ostuni@policlinico.ba.it (A.O.); cosima.battista@policlinico.ba.it (C.B.); 5 Division of Hematology, Fondazione IRCCS Casa Sollievo della Sofferenza, 71013 San Giovanni Rotondo, Italy; n.cascavilla@operapadrepio.it; 6 Division of Hematology, “Monsignor Raffaele Dimiccoli” Hospital, 70051 Barletta, Italy; taniasanteramo@gmail.com (T.M.S.); trials.ematobarletta@gmail.com (G.T.); 7 Division of Hematology, University-Hospital of Foggia, 71122 Foggia, Italy; giantiscia@gmail.com; 8 Division of Hematology, “Cardarelli” Hospital, 86100 Campobasso, Italy; giuliogiordano@hotmail.com; 9 Department of Obstetrics and Gynecology, First I.M. Sechenov Moscow State Medical University, 119991 Moscow, Russia; 10 Department of Obstetrics and Gynecology, University of Foggia, 71122 Foggia, Italy

**Keywords:** thrombotic thrombocytopenic purpura, ADAMTS13, mortality, relapse, outcome

## Abstract

Immune-mediated thrombotic thrombocytopenic purpura is a rare and challenging hematological disease caused by the antibody anti-ADAMTS13. Though the mortality rate has decreased considerably in recent years, fatalities still remain unacceptable. This study aimed at further adding to the existing knowledge of this medical challenge. We enrolled 89 consecutive patients observed in six Italian centers (from 8 August 2013 to 28 May 2021) with a diagnosis of immune-mediated thrombotic thrombocytopenic purpura. Clinical information and blood parameters were collected for all patients. We describe clinical manifestations and laboratory data, possible risk factors and the therapeutic management of first episodes or relapses. A total of 74 first episodes and 19 relapses (median 3 years (interquartile range (IQR): 2–7)) were recorded. Seventy percent of patients enrolled at the first episode showed neurological signs and/or symptoms. All the patients enrolled at the first episode were treated with plasma exchange (median = 12; IQR: 8–19.5) and methylprednisolone (1 mg/kg/day). Rituximab (375 mg/m^2^ weekly for four weeks) and caplacizumab were given to 15 (20.2%) and 2 patients (2.6%), respectively. We observed an overall mortality of 5.4% in the follow-up (median 60 months; IQR: 36.0–103.5). All fatalities occurred after a diagnostic delay. Present data point to the importance of the early detection of factors mostly associated with poor outcomes. It is likely that use of caplacizumab could improve the prognosis in those patients.

## 1. Introduction

Thrombotic thrombocytopenic purpura (TTP) is a rare hematological disease. It is one of the thrombotic microangiopathies (TMAs) characterized by thrombocytopenia, hemolytic anemia and thrombosis in micro-vessels [1]. At the beginning of the 1980, Moake et al. published the first evidence of TTP pathophysiology [2]. They observed that patients with relapsing TTP can show a significant blood accumulation of ultra-large multimers of the von Willebrand factor (VWF)—a platelet adhesive protein. The accumulation of VWF multimers is caused by the deficiency of ADAMTS 13 (A disintegrin and metalloproteinase with thrombospondin motifs 13 (ADAMTS13 Uniprot Q76LX8)), a VWF-cleaving protease. A severe ADAMTS13 deficiency (defined as protease activity below 10% of normal or 10 U/dL), due to mutations within ADAMTS13 gene or specific anti-ADAMTS13 auto-antibodies, induces congenital TTP or immune-mediated TTP (iTTP), respectively [3,4,5,6]. Epidemiological data show that immune-mediated cases account for approximately 95% of all the TTP diagnoses with an incidence rate of 1/250,000 to 1/1,000,000. Furthermore, women are more prone to iTTP [7] and clinical manifestations are quite heterogeneous with a reported higher incidence of neurological symptoms (headache, confusion, coma) [8]. About 50% of iTTP cases are idiopathic, while the remaining cases are apparently associated with conditions such as infections, drugs intake, pregnancy or vaccinations [9,10,11]. Overall, 20–50% patients experience a relapse [12].

A prompt diagnosis is crucial, as plasma exchange (PEX), the mainstay of therapy, reduces mortality rate from about 90% [13] to nearly 15–20% [9]. The advent of caplacizumab—a nanobody neutralizing VWF—has greatly improved the prognosis of iTTP patients, as shown by randomized controlled trials and post-marketing real-world data [14,15,16,17,18]. Indeed, caplacizumab significantly reduces the time to normalize the platelet count and, most importantly, it contributes to decreasing TTP-related deaths and recurrences [19].

In the present paper, we show the clinical and laboratory features of iTTP patients observed between 2013 and 2021 in six Italian centers.

The aim of our study was to broaden current knowledge on the iTTP, with an emphasis on risk factors, disease manifestations and treatments.

## 2. Materials and Methods

### 2.1. Patients and Study Design

We carried out a multicenter prospective study on 89 patients referred to six Italian centers because first episodes of TTP or a relapse from Aug 08, 2013, to May 28, 2021. Before treatment all patients underwent blood sampling to measure ADAMTS13. TTP diagnosis was made if ADAMTS13 activity was less than 10% or 10 U/dL [5]. Clinical and laboratory data were accurately collected from medical records; we paid special attention to information such as recent cancer diagnosis (in the last year preceding the iTTP diagnosis), history of bone marrow or organ transplantation, drugs or hormonal therapies, infectious diseases, co-morbidities, medical history (i.e., diabetes, hypertension, autoimmune diseases, neuropsychiatric behavioral disorders, vaccinations) or recent pregnancy.

We categorized clinical and laboratory manifestations according to the specific physio-pathological features of iTTP, paying specific attention to thrombocytopenia, neurological (motor, cognitive, fatigue, seizures, coma, behavioral alterations), bleeding-related (petechiae, purpura, ecchymosis, hematuria) cardiac (tachycardia, arrhythmia, congestive failure, acute cardiac ischemia), renal (serum creatinine value above 1.3 mg/dL or proteinuria) and gastrointestinal (such as abdominal pain, diarrhea, vomit) signs and symptoms [10]. In addition, we collected the following laboratory data: blood count, hemolysis markers, clotting tests, creatinine, complement components, troponin, routine liver biomarkers and urine parameters. Furthermore, information on treatments (i.e., number of PEX procedures) or adjunctive therapies used in acute events (steroids, Rituximab^®^, caplacizumab) was also collected.

### 2.2. Definitions

Clinical response: sustained normalization of platelet counts above the lower limit of the established reference range (e.g., >150 × 10^9^/L) and of lactate dehydrogenase (LDH) (<1.5 upper limit of normal [ULN]) after cessation of plasma exchange [14,20].

Clinical remission: clinical response after cessation of plasma exchange, maintained for >30 days. Clinical response and clinical remission are associated with stabilization of parameters if end-organ damage is severe or there is an improvement in function with normalization of laboratory parameters [14,20].

Exacerbation: reduction in platelet count to below the lower limit of the established reference range (e.g., <150 × 10^9^/L), an increased LDH level and the need to restart plasma exchange within 30 days of the last plasma exchange (PEX) after a clinical response to PEX [14,20]

Relapse: fall in platelet count to below the lower limit of the established reference range (e.g., <150 × 10^9^/L), with or without clinical symptoms, >30 days after stopping PEX for an acute TTP episode, requiring re-initiation of therapy [14,20].

### 2.3. ADAMTS13 Assay

Blood samples were obtained at the time of hospital admission. In all cases, blood samples were obtained before starting treatment. After centrifugation at 3000× *g* for 10 min, plasma samples were stored at −20 °C until ADAMTS13 measurements. ADAMTS13 activity (normal range: 40.0–130.0 U/dL) was measured using TECHNOZYM^®^ ADAMTS13 Activity ELISA Kit (Technoclone, Vienna, Austria) or FRETSVWF73 (Peptide International, Lexington, KY, USA) assay. Anti-ADAMTS13 IgG concentration was measured with TECHNOZYM^®^ ADAMTS13 INH ELISA (Technoclone, Vienna, Austria), with following reference values: IgG concentration > 15.0 U/mL for positive samples; 12.0–15.0 U/mL for border-line samples; <12.0 U/mL for negative samples), as previously reported [21].

### 2.4. Statistical Analysis

Means, medians (with interquartile range (IQR)) and frequency distributions were calculated for continuous variables. Number and percentages are reported for binary and categorical variables.

### 2.5. Ethics

This study was carried out following recommendations of the Declaration of Helsinki. Ethics approval was obtained and all patients signed a written informed consent.

The study was funded by Italian Minister of Health (RC2001TE11). Research was approved by Ethics Committe of “Casa Sollievo della Sofferenza” Hospital (N. 140/CE).

## 3. Results

In six Italian centers, we observed 89 patients with a confirmed iTTP diagnosis; of these patients, 74 were observed after their first iTTP episode and 15 after the relapse. The enrollment rate was 11.1 patients/year.

### 3.1. Clinical and Laboratory Data

Table 1 shows the demographic and clinical information and relevant laboratory data of patients receiving their first iTTP diagnosis. All showed thrombocytopenia, anemia and hemolysis. In addition, they had ADAMTS13 activity below 10% or 10 U/dL and tested positive for anti-ADAMTS13 antibodies (Table 1). Most were Caucasian (94.5%) with median age of 47.0 years; as expected, a higher prevalence (*n* = 48, 65.5%) of women was observed. With regard to co-morbidities, hypertensive disorders were the most represented (18.2%). Furthermore, two patients had a diagnosis of active pancreatic adenocarcinoma.

With regard to clinical manifestations, most patients (56/74, 75.5%) showed neurological signs and symptoms. Furthermore, renal involvement [(median of glomerular filtration rate = 37 mL/min (Interquartile range (IQR): 28.7–71.5)] was observed in 23 patients (31.0%), whereas bleeding and cardiac manifestations were reported in 32 (43%) and 9 (12%) patients, respectively. Lastly, 34.0% (25/74) experienced three or more clinical manifestations.

In 46 cases, we recorded the concomitant use of several drugs at the time of the iTTP manifestations (Table S1): ten (21.8%) patients were taking antibiotics.

All patients were treated with PEX at the first iTTP episode (median 12; IQR: 8.0–19.5) and methylprednisolone 1 mg/kg/day. Rituximab (RTX) at a standard dose (375 mg/m^2^ weekly for four weeks) was given to 15 patients (20.2%). In addition to PEX, caplacizumab was added in two cases (2.6%), both observed in 2021, when the nanobody was available on the Italian market. Lastly, vincristine treatment was given to two further patients (2.6%).

The median follow-up was 60 months (IQR: 36.0–103.5). Overall, of the 74 patients who enrolled at their first episode, 4 had relapses (5.4%) and were symptomatic with or without a fall in platelet count.

With regard to relapsing episodes (Table 2), we observed 19 episodes (15 patients) with a median interval from the first event of three years (IQR: 2–7). Most relapses (9/19; 47.3%) occurred with bleeding manifestations. Two relapses were associated with coincidental factors: pregnancy in one case and vaccination against SARS-CoV-2 in a male who showed the first symptoms seven days after injection.

Two relapses were observed: one after minor surgery in a 52-year-old male patient who had undergone excision of the lipoma of the fossa femoralis and the other after the administration of intravenous contrast medium. In this last case, the episode occurred in a 75-year-old male in follow-up for pancreatic adenocarcinoma, who showed thrombocytopenia (30 × 10^9^/L) two days after the CT scan.

We calculated a median of 15 PEX sessions (IQR: 6–21) in the entire group of relapses. In all relapses, steroids were always prescribed, whereas a standard dose of RTX (375 mg/m^2^ weekly for four weeks) was given to three patients. None of the patients with relapse had followed a prevention treatment scheme.

The only observed exacerbation was diagnosed in a 29-year-old woman taking anti-inflammatory drugs without co-morbidities. She had been discharged with a platelet count of 253 (10^9^/L). After five days, she required further hospitalization, as her platelet count dropped to 7 (10^9^/L) with an ADAMTS13 activity of 1 U/dL. She underwent additional eight PEX and successfully underwent RTX infusions (375 mg/m^2^ weekly for four weeks).

Lastly, during the entire period of follow-up, one patient (1.3%) was diagnosed with ischemic stroke three years after the iTTP acute episode and three further patients (3/74; 4.0%) were diagnosed with autoimmune disorders (Sjogren syndrome, undifferentiated connective tissue disease and autoimmune hypothyroidism).

### 3.2. Mortality

In the entire observation period, we observed seven deaths, two of which were not related to iTTP; therefore, five (5.6%) fatalities occurred during the first episode of iTTP (*n* = 4) or relapse (*n* = 1).

Overall, the length of stay was near or lower than the 25th percentile (9.5 days). Fatalities were observed after a median of 9.5 days (IQR: 3.0–10.7). The number of PEX procedures in fatalities was nearly or lower than the 25th percentile (25th percentile = 7.5).

The first case (occurred at the first day of hospitalization) was recorded in a 29-year-old male, who postponed emergency admission and was thus diagnosed one week after the onset of a dysarthria. He died before any treatment could be initiated.

With regard to the other four fatalities, a woman who presented with impaired kidney functions (estimated Glomerular Filtration Rate (eGFR) = 21) and a cardiac involvement (troponin concentration = 64.9 ng/mL; normal range: 0.000–0.045 ng/mL) died on the 11th day of hospitalization. She received the diagnosis two days after disease onset; PEX as well as steroids were started at diagnosis and the first RTX dose was administered on the ninth day of hospitalization.

The third death that occurred (after 15 days of hospitalization) was a woman chronically treated with antidepressants who showed aphasia, hematoma in one leg and abdominal pain at the iTTP diagnosis (made three days after the disease onset). Laboratory investigations showed impaired kidney function (eGFR = 21) and cardiac involvement (troponin concentration = 1.2 ng/L; normal range: 0.000–0.045 ng/mL). She first started PEX and steroids; after 3 and 13 days, respectively, she was also administered RTX. The fourth fatality occurred seven days after the diagnosis in a 54-year-old woman who showed hemiparesis and aphasia at the disease presentation. She showed non-neutralizing anti-ADAMTS13 antibodies and abnormal troponin levels (troponin concentration = 0.118 ng/mL; normal range: 0.000–0.045 ng/mL). After the iTTP diagnosis—four days following disease onset—she started PEX and steroids; the first dose of RTX was also administered six days after the diagnosis. The fifth fatality occurred in a 62-year-old woman who was hospitalized because of a relapse, which occurred 18 months after the first iTTP. At relapse, she displayed neurological and bleeding manifestations, as well as renal involvement in the apparent absence of triggers. After the hospital admission, she was diagnosed with abnormal cardiac re-polarization and died after two days of hospitalization.

## 4. Discussion

In this article, we describe a series of iTTP cases recruited in six Italian centers during a wide frame of time. We focused especially on the most relevant clinically outcomes, laboratory findings and management.

Drug intake was associated with 64% of first episodes; this confirms the speculation that TMA, including TTP, occurs through either a toxicological or an immunological mechanism [22]. For this reason, accurate pharmacologic data collection deserves special attention from physicians. Indeed, they need to be aware that some drugs can induce TMA in order to identify at risk patients, especially among those who take several pharmacologic treatments.

Our study confirms the heterogeneity of iTTP; indeed, our patients showed a variety of clinical manifestations, although the vast majority of patients show neurological symptoms, as often reported in [23,24,25]. We found that factors such as neurological or cardiac damage, as well as a delay in diagnosis, are responsible for a worse prognosis. As expected, women are largely represented (65.5%), in agreement with data from previous studies (68.0% to 75%) [24,26,27,28,29,30].

There is a substantial difference in reporting kidney impairment in iTTP. In fact, some authors recorded kidney impairment in 50% to 76.0% of cases [29,30,31,32], whereas others reported lower percentages [28], similar to those we found (14.0%). This heterogeneity can be explained by a different cut-off for creatinine and, occasionally, the scarce attention to kidney involvement. Despite the relatively high number of bleeding manifestations (40%), no life-threatening or fatal events occurred, in agreement with previous reports [26].

Relapsing episodes were mostly associated with neurological manifestations and most occurred within two years after the first event, as previously described [33]. We observed a relapse rate of 1.9% per month, which is similar to what has been previously reported (2.6% per month) [31]. However, this information needs to be interpreted with caution, due to the possible influence of therapies used and the follow-up duration. It is likely that strict and careful follow-up programs and patients’ disease awareness could make the manifestations of relapse less clinically relevant than first episodes.

In the present study, the overall mortality rate was 5.4%; this figure is slightly lower than that reported in regional UK TTP registry (8.5%) [32] and in other registries [24,30,34,35,36]. In available registries, the age of patients was similar to that found in those described in the current study, although in some cases the sample size was larger and the races were different. Large registries recruited more than 150 patients; furthermore, patients were mainly represented by Afro-Caribbean or Japanese ancestries [9]. We can speculate that race can have impact on the iTTP severity and, in turn, on mortality.

In agreement with data from the Spanish registry, patients in our study with a fatal outcome showed severe neurological manifestations and a delayed diagnosis. Indeed, it has been previously documented that poor neurological conditions are predictors of mortality [22,36,37,38]. A possible limitation of our study was that most patients described here were recruited before 2020, in the pre-caplacizumab era, suggesting a potential risk for bias. However, the figure of mortality is not different from that reported in the Spanish registry [36] or the Milan TTP registry [29]. Furthermore, we carefully analyzed the approach to treatment by the center and year without identifying any substantial differences across the years. We highlighted that all fatalities occurred after a diagnostic delay; these data, once again, point to the importance of the early detection of factors mostly associated with poor outcomes. It is likely that use of caplacizumab could have improved the prognosis in those patients. Unfortunately, most cases shown here were recruited before the availability of this drug in centers where these patients were hospitalized. Another limitation of the study lies in the relatively small sample size and, in turn, a small number of events.

It is known that a non-response to the first few PEX is a strong indicator of mortality [39,40]; on the other hand, our patients show good clinical outcomes independently of the PEX number, in agreement with previous studies [27,41]. Noteworthy, in only 4 out of 19 relapses (21%) the platelet number normalized with less than 5 PEX. This is consistent with suggestions from Scully et al. [42] and the recent International Group consensus, which stressed that persistent thrombocytopenia after 5 PEX is the most relevant indicator of refractoriness [20,43]. It is likely that a systematic association of anti-VWF therapy and corticosteroids and rituximab will allow for reducing the incidence of refractory disease.

## 5. Conclusions

As for any other rare disease, TTP shows high variability with regard to features and outcomes. This may reflect differences in management in addition to genetic background.

Results from the present cohort confirm and extend some recent findings from other registries. Furthermore, the present findings are helpful in improving treatment strategies and increasing physicians’ awareness of the disease and the need for early diagnosis and the most appropriate treatments. An accurate and more and more personalized therapeutic approach, i.e., combination of standard and novel therapies, will further decrease mortality, severe clinical manifestations and thrombotic sequelae.

## Figures and Tables

**Table 1:** Demographic/clinical information and laboratory data of patients experiencing first TTP episode, *n* = 74.

Variables	Values
Demographic/clinical information	
Age at diagnosis, median (IQR)	47 (39–61)
Female patients, *n* (%)	48.0 (65.5)
Race	
White, *n* (%)	69.0 (94.5)
African, *n* (%)	4.0 (5.5)
Clinical manifestations	
Neurological	58 (78.0)
Bleeding, *n* (%)	32 (43.0)
Renal, *n* (%)	23 (31.0)
Gastrointestinal, *n* (%)	20 (27)
Cardiac, *n* (%)	9 (12.0)
Co-morbidities and medical history	
Hypertension, *n* (%)	13.0 (17.5)
Infectious diseases *, *n* (%)	12.0 (16.2)
Autoimmune diseases ^, *n* (%)	9.0 (12.1)
Vaccination**^§^**, *n* (%)	7.0 (9.5)
Major depressive disorders, *n* (%)	4.0 (5.4)
Diabetes, *n* (%)	4.0 (5.4)
Cancer, *n* (%)	2.0 (2.7)
History of VTE°, *n* (%)	1.0 (1.3)
Deaths, *n* (%)	4.0 (5.4)
Relapses, *n* (%)	4.0 (5.4)
Remissions, *n* (%)	69.0 (94.5)
Treatments	
PEX, median (IQR)	12 (8.0–19.5)
RTX, *n* (%)	15 (20.2)
Caplacizumab, *n* (%)	2 (2.6)
Laboratory data	
ADAMTS13 activity, median (IQR), IU/dL	1.0 (1.0–2.0) (n.r. 40.0–130.0)
ADAMTS13 inhibitor titer, median (IQR), Bethesda unit	2.0 (1.3–16.0) (n.r. < 0.4)
Anti-ADAMTS13 IgG, median (IQR), U/mL	71.0 (31.0–91.0) (n.r. < 15.0)
Platelet count, median (IQR), 10^9^/L	11.0 (7.0–20.5) (n.r. 150.0–350.0)
Hemoglobin, median (IQR), g/dL	8.1 (7.0–9.9) (n.r. 12.0–15.0)
Troponin, median (IQR), ng/mL	0.8 (0.1–4.8) (n.r. 0.00–0.045)
Reticulocytes, median (IQR), %	6.1 (3.7–8.9) (n.r. 0.6–2.7)
Lactate dehydrogenase (LDH), median (IQR), IU/L	899.5 (633.5–1624.0) (n.r. 122.0–222.0)
Creatinine, median (IQR), mg/dL	1.1 (0.8–1.3) (n.r. 0.6–1.2)
Alanine transaminase (ALT), median (IQR), IU/mL	27.0 (20.0–44.0) (n.r. 7.0–55.0)
Aspartate transaminase (AST), median (IQR), IU/mL	37.0 (27.0–55.0) (n.r. 8.0–40.0)
Prothrombin time, median (IQR), INR	1.0 (0.9–1.1) (n.r. 0.8–1.2)
Activated partial thromboplastin time (aPTT), median (IQR), ratio	0.9 (0.8–1.0) (n.r. 0.8–1.2)

IQR: interquartile range; * Flu-like syndrome (4), higher respiratory tract (4), urinary tract (2), gastrointestinal tract (1), hepatitis C (1). ^ A total of 6 additional patients showed isolated anti-smooth muscle antibodies or antineutrophil cytoplasmic antibodies; ^§^ all against hepatitis B virus; °VTE: Venous thromboembolism. n.r.: normal range.

**Table 2:** Patients with relapses: demographic and clinical information.

Age, Median (IQR)	34 (31–48)
Sex, F/M	14/5
Coincidental conditions, n (%)	Pregnancy, 1 (5.2)
	Surgery, 1 (5.2)
	Vaccination anti-SARS-CoV-2, 1 (5.2)
	Unknown, 15 (79.2)
Clinical signs and symptoms, n (%)	Bleeding, 9 (47.3)
	Neurological, 6 (31.5)
	Cardiac, 2 (10.5)
Treatment, n (%)	PEX, 19 (100)
	Steroids, 19 (100)
	Rituximab, 3 (27) *
Outcome, n (%)	Remissions, 18 (94.7)
	Deaths, 1 (5.3)

* Information available for 11 patients.

## Data Availability

The data presented in this study are available on request from the corresponding author.

## Supplementary Materials

The following are available online at https://www.mdpi.com/article/10.3390/jcm10235702/s1, Table S1: Drugs recorded in the study population of patients experiencing first iTTP episode, *n* = 74.
