# Supplementary material for: Focus on Key Issues in Immune Thrombotic Thrombocytopenic Purpura: Italian Experience of Six Centers
Source: J Clin Med. 2021 Dec 4;10(23):5702. doi: 10.3390/jcm10235702 (PMC8658151; doi:10.3390/jcm10235702)
Supplement: Supplementary file 1 [file jcm-10-05702-s001.zip › jcm-1471560-supplementary.pdf]

**Supplementary Table S1.** Drugs recorded in the study population of patients experiencing first iTTP episode, $n$ = 74.

| **Drugs** | **Values** |
|---|---|
| Antibiotics, $n$ (%) | 10 (13.5) |
| Statins, $n$ (%) | 6 (8.1) |
| Beta blockers, $n$ (%) | 5 (6.7) |
| Calcium channel blockers, $n$ (%) | 4 (5.4) |
| Angiotensin-converting enzyme (ACE) inhibitors, $n$ (%) | 4 (5.4) |
| Sartans, $n$ (%) | 4 (5.4) |
| Anti-depressants, $n$ (%) | 4 (5.4) |
| Oral Contraceptives, $n$ (%) | 3 (4.0) |
| NSAIDs, $n$ (%) | 2 (2.7) |
| Immunosuppressants, $n$ (%) | 1 (1.3) |
| Chemotherapy, $n$ (%) | 1 (1.3) |
| Anti-malarial, $n$ (%) | 1 (1.3) |
| Clopidogrel, $n$ (%) | 1 (1.3) |

NSAIDs: Non-Steroidal Anti-Inflammatory Drugs.
